# Building work engagement: A systematic review and meta‐analysis investigating the effectiveness of work engagement interventions

**DOI:** 10.1002/job.2167

**Published:** 2016-12-13

**Authors:** Caroline Knight, Malcolm Patterson, Jeremy Dawson

**Affiliations:** ^1^ Institute of Work Psychology University of Sheffield Management School Sheffield South Yorkshire UK

**Keywords:** work engagement, interventions, meta‐analysis, systematic review, intervention implementation

## Abstract

Low work engagement may contribute towards decreased well‐being and work performance. Evaluating, boosting and sustaining work engagement are therefore of interest to many organisations. However, the evidence on which to base interventions has not yet been synthesised. A systematic review with meta‐analysis was conducted to assess the evidence for the effectiveness of work engagement interventions. A systematic literature search identified controlled workplace interventions employing a validated measure of work engagement. Most used the Utrecht Work Engagement Scale (UWES). Studies containing the relevant quantitative data underwent random‐effects meta‐analyses. Results were assessed for homogeneity, systematic sampling error, publication bias and quality. Twenty studies met the inclusion criteria and were categorised into four types of interventions: (i) personal resource building; (ii) job resource building; (iii) leadership training; and (iv) health promotion. The overall effect on work engagement was small, but positive, *k* = 14, Hedges *g* = 0.29, 95%‐CI = 0.12–0.46. Moderator analyses revealed a significant result for intervention style, with a medium to large effect for group interventions. Heterogeneity between the studies was high, and the success of implementation varied. More studies are needed, and researchers are encouraged to collaborate closely with organisations to design interventions appropriate to individual contexts and settings, and include evaluations of intervention implementation. © 2016 The Authors. *Journal of Organizational Behavior* published by John Wiley & Sons, Ltd.

## Introduction

Work engagement is currently a popular topic within many organisations, given its association with employee well‐being and performance (e.g. Christian, Garza, & Slaughter, 2011; Halbesleben, 2010). Evaluating, boosting and sustaining work engagement are therefore a prime concern of many organisations, and many studies have investigated the possible antecedents and consequences of engagement (e.g. Halbesleben, 2010; Crawford, LePine, & Rich, 2010), leading researchers to consider the field sufficiently well developed to warrant the development and testing of work engagement interventions (e.g. Leiter & Maslach, 2010). However, the evidence on which to base interventions is limited, although a variety of intervention studies are emerging (Biggs, Brough, & Barbour, 2014; Ouweneel, Le Blanc, & Schaufeli, 2013). No study has yet assessed the effectiveness of these interventions; however, it is hoped that doing so will stimulate debate and direct future research and practice. The aim of this study is therefore to conduct a narrative systematic review and meta‐analysis of the evidence for the effectiveness of controlled work engagement interventions.

### Work engagement

Kahn (1990) originally pioneered the concept of employee engagement, proposing that engaged employees are physically, cognitively and emotionally involved in their work roles, and experience a sense of meaning (reward for investing in role performance), psychological safety (a sense of trust and security at work) and availability (a sense of having the physical and psychological resources necessary for the job). Saks (2006) developed this view by distinguishing between job and organisational engagement to reflect the different roles of employees. Maslach and Leiter (1997) approached engagement from the field of burnout, characterising it in terms of high energy, involvement and efficacy, the polar opposite of burnout (exhaustion, cynicism and inefficacy), and therefore measurable using the Maslach Burnout Inventory (MBI; Maslach & Jackson, 1981). Schaufeli, Salanova, Gonzalez‐Roma, and Bakker (2002) refuted this, arguing that while engagement is the positive antipode to burnout, it is a separate, distinct concept, and therefore cannot be measured on a burnout scale. They defined work engagement as a state of mind characterised by vigour, dedication and absorption (Schaufeli et al., 2002), and developed the Utrecht Work Engagement Scale (UWES) to measure it. Vigour refers to high energy and mental resilience while working, dedication to being intensely involved in work tasks and experiencing an associated sense of significance, enthusiasm, and challenge, and absorption to a state of full concentration on work and positive engrossment in it.

Schaufeli et al. (2002) view of engagement as a distinct concept from burnout was contested by Cole, Walter, Bedeian, and O'Boyle (2012), who found in their meta‐analysis, involving 50 independent samples, that burnout and engagement were highly correlated, similarly associated with correlates, and that controlling for burnout reduced the effect sizes of engagement substantially. They concluded that whether burnout and engagement can be viewed as separate dimensions was questionable.

Further academic (e.g. Crawford et al., 2010; May, Gilson, & Harter, 2004), and lay and practitioner (e.g. MacLeod & Clarke, 2009; Robertson‐Smith & Markwick, 2009) definitions and measures of engagement exist, and scholars have also questioned the existence of engagement, arguing that it is redundant with other, established job attitudes such as job satisfaction, organisational commitment and job involvement (for a good review, see Macey & Schneider, 2008; see also Byrne, Peters, & Weston, 2016; Christian et al., 2011). Questions have also arisen over the factorial validity of the UWES, with some studies suggesting that a three‐factor model is superior to a one factor, unidimensional model (e.g. Bakker & Demerouti, 2008; Schaufeli, Bakker, & Salanova, 2006), and others suggesting that the three factor structure is ambiguous (e.g. Sonnentag, 2003), or that the models are equivalent (Hallberg & Schaufeli, 2006). The field is clearly divided over the meaning of engagement and how best to measure it. Nevertheless, Schaufeli et al. (2002) perspective appears to be the most popular and well researched to date (Hakanan & Roodt, 2010), and tends to underlie engagement interventions.

The key model underlying Schaufeli et al. (2002) perspective is the Job Demands–Resources model (JD‐R; Bakker & Demerouti, 2008; Bakker & Demerouti, 2007). This proposes that work engagement is driven, either independently or together, by both job and personal resources. Job resources refer to physical, social or organisational aspects of the job (e.g. feedback, social support, development opportunities) that can reduce job demands (e.g. workload, emotional and cognitive demands), help employees to achieve work goals, and stimulate personal learning and development. Personal resources refer to ‘positive self‐evaluations that are linked to resiliency and refer to individuals' sense of their ability to control and impact upon their environment successfully’ (Bakker & Demerouti, 2008 p.5). These include self‐esteem, self‐efficacy, resilience and optimism. The motivating potential of job and personal resources are proposed to lead to positive individual and organisational outcomes such as work engagement, well‐being and performance, whereas few resources and high work demands are proposed to lead to poor health outcomes, such as burnout, stress and depression, as well as turnover, sickness absence and poor performance. This model has been supported by numerous studies (e.g. Xanthopoulou, Bakker, Demerouti, & Schaufeli, 2009; Hakanen, Bakker, & Demerouti, 2005; Simbula, Guglielmi, & Schaufeli, 2011), including meta‐analyses (Crawford et al., 2010; Halbesleben, 2010; Nahrgang, Morgeson, & Hofmann, 2011), all of which have served to advance the model and work engagement theory.

Bakker and Demerouti (2007) posit that four specific processes underlie the motivational potential of job and personal resources. These are: (i) broaden‐and‐build theory (Fredrickson, 2001), which proposes that positive emotions enable employees to increase their personal resources by widening the spectrum of thoughts and actions that come to mind; (ii) the experience of better health, which is proposed to enable employees to focus all their resources on the job; (iii) job crafting, which refers to employees creating their own opportunities and resources; and (iv) emotional contagion theory, which suggests that employees transfer engagement to others, indirectly improving team engagement and performance (Bakker, Van Emmerik & Euwema, 2006, 2009). According to Bakker (2011), these processes create a positive gain spiral of engagement over time.

### Work engagement interventions

We are currently aware of no other reviews of work engagement interventions. An initial scoping review, 1A scoping review is a relatively new term which is typically defined as a rapid, non‐systematic review of the literature which attempts to provide an overview of the key literature in an area (Mays, Roberts, & Popay, 2001). Arksey and O'Malley (2005) suggest that such a review is pertinent for determining the potential of conducting a full systematic review, as in our case. Other, similar, definitions exist (for an overview, see Levac, Colquhoun, & O'Brien, 2010). however, revealed the emergence of several interventions since 2010. Almost all of these adopted Schaufeli et al. (2002) conceptualisation of work engagement, although large heterogeneity between the studies was revealed in terms of research design, participant characteristics, content, duration and the organisation's location and industry. Whether studies measured overall work engagement, or one or other of the three sub‐components, also varied, which has implications for the ability to group studies together for a meta‐analysis. We identified four types of intervention 2Because no previous classification system exists, intervention categories were identified through in‐depth coding of the studies by two expert researchers (see Method).: (i) personal resource building interventions; (ii) job resource building interventions; (iii) leadership training interventions; and (iv) health promoting interventions.

Personal resource building interventions focus on increasing individuals' self‐perceived positive attributes and strengths, often by developing self‐efficacy, resilience or optimism (e.g. Ouweneel et al., 2013; Vuori, Toppinen‐Tanner, & Mutanen, 2012). Employees with high levels of personal resources are thought to positively appraise their ability to meet their work demands, believe in good outcomes and believe they can satisfy their needs by engaging fully in their organisational roles. In accordance with the Job Demands–Resources Model (JD‐R; Bakker & Demerouti, 2007), personal resources may directly or indirectly lead to work engagement, in the latter case by buffering against the negative effects of perceived job demands. The results of personal resource building interventions on work engagement have been mixed. For example, Ouweneel et al. (2013) observed a positive, significant effect for those who were initially low in engagement only, whereas Calitz (2010) observed positive, significant effects for both engagement sub‐components measured, vigour and dedication, and Sodani, Yadigari, Shfia‐Abadi, and Mohammadi (2011) found significant effects of all three sub‐components. Others found no effects at all, however (e.g. Chen, Westman, & Eden, 2009; Vuori et al., 2012).

Job resource building interventions focus on increasing resources in the work environment such as autonomy, social support and feedback (e.g. Naruse et al., 2014), and are predicted to lead to work engagement, well‐being and performance (Bakker & Demerouti, 2008). According to the motivational process underlying the JD‐R Model, job resources intrinsically motivate employees by stimulating growth, learning and development, satisfying basic human needs for autonomy, relatedness and competence (Deci & Ryan, 2000), or extrinsically motivate employees by providing the means by which work goals can be accomplished. Furthermore, Conservation of Resources (COR) theory (Hobfoll, 2002) suggests that employees will seek to retain and increase resources they value; hence, those with more resources are less likely to experience resource loss and more likely to seek further resources. The initial scoping review suggested that job resource building interventions on work engagement have so far failed to find any significant effects; however, studies have demonstrated positive, non‐significant, increases in work engagement (e.g. Naruse et al., 2014), or its sub‐components (e.g. Cifre, Salanova, & Rodriguez‐Sanchez, 2011).

Leadership training interventions involve knowledge and skill building workshops for managers and measure work engagement in their direct employees (e.g. Rigotti et al., 2014). The assumption is that increasing the knowledge and skills of managers will increase employees' perceived sense of job resources, motivating them to engage in their work according to the motivational hypothesis of the JD‐R Model. Results of leadership interventions on work engagement have been mixed. For example, Biggs et al. (2014) found positive, significant effects on work engagement when their intervention was mediated by employees' perceptions of work–culture support and strategic alignment, whereas Rigotti et al. (2014) found a borderline significant effect in their German study but no effect in their Swedish study, despite implementing very similar interventions in both locations. These results point to the potential importance of context, and tailoring interventions to individual circumstances and organisational needs (Briner & Walshe, 2015; Nielsen, Taris, & Cox, 2010).

Health promoting interventions encourage employees to adopt and sustain healthier lifestyles and reduce and manage stress. For example, the physiological effects of exercise may increase well‐being and work engagement and reduce stress, burnout, poor mental health, sickness absenteeism, and presenteeism (Strijk, Proper, Van Mechelen, & Van der Beek, 2013). The positive emotions which can follow exercise may widen individuals' range of thoughts and actions in accordance with broaden‐and‐build theory (Fredrickson, 2001), and enable personal resources to be built. Mindfulness training may work similarly, by increasing resilience and self‐esteem through increased non‐judgemental acceptance of thoughts, feelings and bodily sensations (Van Berkel, Boot, Proper, Bongers, & Van der Beek, 2014). Again, health promoting interventions have demonstrated mixed results with regards to work engagement. For example, while some have revealed no effects (e.g. Hengel, Blatter, Joling, van der Beek, & Bongers, 2012; Van Berkel et al., 2014), one observed a small but significant effect at both three and six months (Imamura et al., 2015), and one observed significant effects on the vigour sub‐component for a group which was highly compliant with a yoga programme (Strijk et al., 2013).

Two of the studies reported above demonstrated significant effects on a subgroup. Ouweneel et al. (2013) results suggest that it may be worth targeting interventions towards those who are most in need of them, that is, those who report low engagement scores. This is in keeping with recommendations to assess the need for interventions prior to implementing them (e.g. Briner & Walshe, 2015). Strijk et al.'s (2013) results suggest that the successful implementation of interventions, including the compliance of participants, may be crucial to their effectiveness. This indicates that determining the readiness to change of organisations and participants is important, as advocated by Nielsen, Randall, Holten, and González (2010). They highlight that without the strong support of senior management and a perceived need for change by participants, change is unlikely to occur. For a deeper discussion of issues concerning the planning, implementation and evaluation of intervention studies, which is beyond the scope of this paper, see Briner and Walshe (2015), or Nielsen, Randall et al. (2010).

The steady emergence of a number of work engagement interventions, and the mixed results which these have demonstrated, suggests that a review assessing the effectiveness of interventions is timely. Such a review would help direct future research and contribute towards the developing evidence‐base. This study aims to assess the effectiveness of controlled work engagement interventions by conducting a systematic search of the literature and statistically meta‐analysing the results of those which are able to be included (see Method for inclusion criteria). The first question addressed by this review is therefore as follows:

Research question 1.: *Are work engagement interventions effective?*

Given the variety of interventions emerging, and the different mechanisms by which each is purported to increase work engagement, it is possible that intervention effectiveness will be moderated by intervention type. A recent meta‐analysis in the related field of burnout found that cognitive behavioural techniques were more effective for decreasing exhaustion (a sub‐component of burnout) than other types of intervention (Maricuţoiu, Sava, & Butta, 2016). Another review also found significant differences in the effectiveness of different types of interventions in reducing stress, with cognitive‐behavioural programmes demonstrating the largest effect (Richardson & Rothstein, 2008). The following question will therefore also be explored:

Research question 2.: *Is intervention type (i.e. whether interventions are personal resource building, job resource building, leadership training programmes, or health promotion) associated with intervention effectiveness?*

## Method

### Search strategy

An extensive systematic literature search was conducted between May 2014 and May 2015, in relevant databases, in accordance with current standards outlined in The Cochrane Collaboration's (Higgins & Green, 2011) guidelines for systematic reviews. 3The Cochrane Collaboration is an independent, non‐profit, non‐governmental organisation which exists solely to conduct rigorous systematic reviews and meta‐analyses to inform decision making in healthcare. Contributors are often world leaders in their field (e.g. medicine, health policy, research methodology), and many come from the most respected academic and medical institutions worldwide (for more information, see www.cochrane.org). Briefly, their guidelines state that systematic reviews should contain clear objectives, an explicit, reproducible methodology, attempt to identify all studies meeting inclusion criteria, include an assessment of the validity of studies (e.g. risk of bias) and systematically present the findings. We have attempted to meet these standards in our systematic review. This revealed 726 hits overall. Published studies were identified through searching MEDLINE, Web of Science, Scopus and Google Scholar. Unpublished studies were identified through searching Proquest Digital Dissertations and Theses (PDD), Trove and Thesis Canada Portal, key repositories for Master's and PhD dissertations which cover the UK, Australia and Canada, respectively.

Search strategies were developed for each database specifically, to accommodate variation in the search methods employed by each one (see Supplemental file 1). Key terms included ‘work engagement’, ‘intervention’, ‘group’, ‘individual’, ‘online’ and ‘web’. Manual searches of relevant books (e.g. Albrecht, 2010; Bakker & Leiter, 2010), and key author websites (e.g., Schaufeli, Bakker) were also conducted.

### Criteria for inclusion

Each study was required to meet the following criteria to be included in the meta‐analyses: (i) report a controlled intervention (any intervention with some form of control, comparison or referent group, whether randomised or non‐randomised, equivalent or non‐equivalent, waiting‐list or non‐waiting‐list) conducted with employees of an organisation, whether group based, individual or online; (ii) contain a measure of engagement with evidence of reliability and validity; and (iii) contain a pre and post measure of engagement for both the control/referent and intervention groups, and all other necessary results required to conduct a meta‐analysis (see statistical procedures). In order to capture as many interventions as possible, no restriction was placed on the conceptualisation of engagement adopted or engagement measure used. In reality, only one study was included which had not used the UWES (Aikens et al., 2014), and similar to the UWES, this measure incorporated a cognitive, emotional and behavioural component. Although an unintended consequence, this allowed the included studies to be synthesised more meaningfully than if various conceptualisations and measures of engagement had been used.

**Figure 1:**
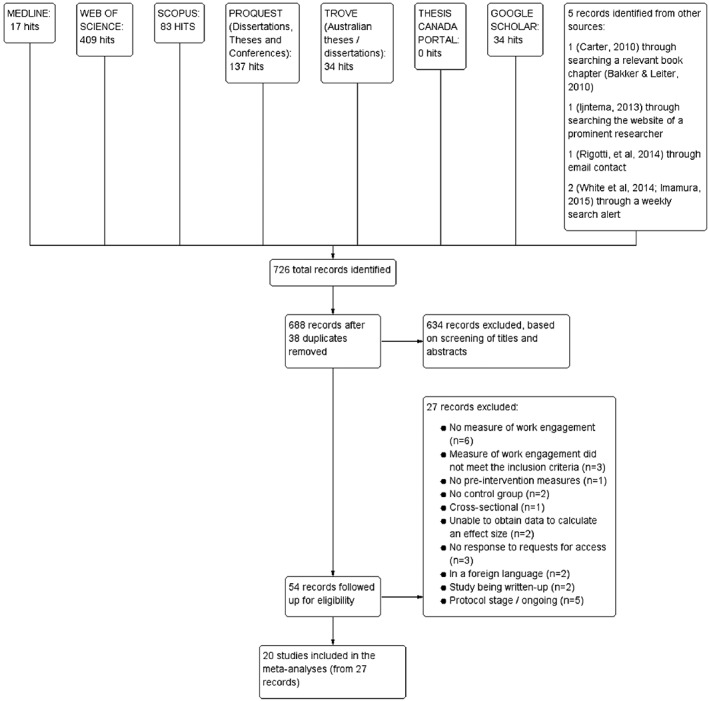
A flow diagram of the systematic literature search results, indicating databases searched, number of hits and reasons for study exclusion

Following the systematic search, duplicates were removed and the remaining titles and abstracts screened for inclusion. The full texts of articles passing this initial screening were obtained and authors contacted for access or more information where necessary. Consequently, 20 studies, represented by 27 articles, were included in the meta‐analyses. The vast majority of excluded studies were not controlled intervention studies or did not measure engagement (Figure 1).

### Coding of the studies

Characteristics of the studies were double coded by one of the authors and an independent researcher working in a related field, according to a specially developed coding guide (see Supplemental file 2). Demographic information extracted were participant numbers and the mean participant age and gender distribution at baseline. Study characteristics included the author details and document date, type of document found (published article, thesis, or grey literature), country of intervention location, the industry of the organisation, whether it was public (owned, controlled and funded by the government) or private, the core components of the intervention (e.g. workshops, coaching, assignments), intervention type (as defined in the introduction) and intervention style (whether or not the intervention was group or individually orientated, a combined group and individual intervention, or conducted purely online). Other particulars included intervention duration, design (e.g. number of groups, presence of randomisation, presence of a control/referent group), measure of work engagement used, response rates, attrition rates, outcomes (i.e. results for which overall work engagement, vigour, dedication and absorption were reported), whether or not results were adjusted for covariates such as age and gender, and key conclusions. Factors highlighted by authors which may have affected the implementation and success of the intervention were also noted, such as lack of participation, mergers, redundancies and economic adversity. These were intended to inform a discussion of the results and provide deeper insight into the results obtained.

Categories were generated for intervention type and style based on the themes emerging from the data itself, and took into consideration the differences and similarities between the interventions. For example, when assessing a study for intervention type, particular attention was paid to how authors portrayed the aims of their interventions, as well as to the characteristics of interventions. For instance, if an author primarily focused discussion on vitality and health (e.g. Strijk et al., 2013), and the intervention was presented as a health promotion programme, the intervention was classified as such. If an author primarily focused discussion on personal resources such as self‐esteem (e.g. Ouweneel et al., 2013) and presented their intervention as one to build personal resources, the intervention was classified as a resource building intervention.

The agreement rate between the two coders was assessed using Cohen's Kappa (Cohen, 1960), which indicates the percentage agreement over and above agreement expected by chance. Values lie between −1.00 and +1.00, with 0 indicating chance agreement, +1.00, perfect agreement, and −1.00, perfect disagreement. Values between 0.40 and 0.59 suggest fair agreement, 0.60–0.74, good agreement and >0.75, excellent agreement (Orwin, 1994). All of the agreement rates were above 0.60, except for one, which was 0.44 (for intervention style). Many were above 0.75 and approached 100%. All differences were resolved by discussion and consultation with a third expert where necessary (another author). Following this process, consensus rates reached 100% for every single piece of data extracted.

### Statistical procedure

The meta‐analyses were computed using the statistical package, Comprehensive Meta‐Analysis (Borenstein, Hedges', Higgins, & Rothstein, 2011). Standardised differences in means (Hedges *g* 4Hedges *g* is considered less biased than the traditionally computed effect size, Cohen's *d*, when small sample sizes are involved (Johnson & Eagly, 2000), as in the present study.) and their 95% confidence intervals were computed for each study. Hedges *g* expresses the difference between the control and intervention group means, divided by their pooled standard deviations. A positive value indicates that the intervention had a positive effect on work engagement, with values between 0.3 and 0.5 representing a medium effect (Cohen, 1988). Hedges *g* was computed using pre‐post intervention means, their respective standard deviations (*SD*) and pre‐post correlations. Where pre‐post correlations were unavailable, a conservative estimate (*r* = 0.7) was adopted in accordance with Rosenthal (1991) recommendation and previous meta‐analyses in related fields (e.g. Khoury et al., 2013). In the absence of means and *SD*s, alternative statistics such as *F* or *p* values were sought. Where necessary, the corresponding author was contacted to obtain the required results.

The mean effect size for a group of studies was calculated by pooling individual study effect sizes according to a random effects approach. This approach assumes that studies come from different populations with different average effect sizes and is appropriate when studies are not identical (Field & Gillett, 2010), as in the present study where studies vary according to factors such as occupational setting, nationality and intervention type. Results were statistically assessed for heterogeneity using the chi‐squared statistic, Cochran's *Q*, and *I^2^*. *I^2^* is not affected by low statistical power, and a value of 25% suggests that heterogeneity is low, 50%, that it is moderate, and 75%, that it is high (Higgins, Thompson, Deeks, & Altman, 2003). High heterogeneity within a group of studies suggests the presence of moderators. To explore the impact of potential moderators on the results, the average effect size for each level of a moderator (e.g. for all four intervention types) was computed and the *Q* statistic was calculated. A significant difference between the effect sizes for each group indicates the presence of a moderator. Results were also assessed for systematic sampling error, sensitivity analyses and publication bias, via Rosenthal's (1991) Fail‐Safe N and Duval and Tweedie's (2000) Trim and Fill method.

The quality of the studies was assessed using ‘Risk of Bias’ tool which assesses studies as ‘low risk’, ‘unclear risk’ or ‘high risk’ according to five criteria: (i) selection bias; (ii) allocation concealment; (iii) performance bias; (iv) detection bias; and (v) attrition bias (Higgins, Altman, & Sterne, 2011). In general terms, a study is rated as ‘high risk’ on a criterion if there is evidence of bias which is likely to substantially affect the results or conclusions drawn (Higgins et al., 2011). For example, an intervention study claiming to be randomised is rated at low risk of selection bias if the authors have described a random method of allocating participants to the intervention (e.g. using a computerised random number generator, coin tossing, throwing dice), at high risk if a non‐random method has been used (e.g. using odd and even dates of birth, or the availability or location of the participant), and at unclear risk if there is insufficient information to make a judgement. A study rated as ‘high risk’ in at least one of the areas is given an assessment of ‘high risk’ overall. Each study was again double coded, and initial Cohen's Kappa agreement rates reached 0.52 or above, with two reaching 100% (for assessments of performance bias, that is, whether blinding of participants and personnel occurred, and overall summary judgements). Again, all discrepancies were resolved by discussion, resulting in 100% agreement for all risk of bias judgements.

We acknowledge that assessing the quality of studies based on five criteria only is arguably limiting, and omits an assessment of how well the intervention was implemented and adhered to. These factors can have a large impact on the effectiveness of interventions and have led some researchers to strongly promote their inclusion in the evaluation of organisational interventions as a matter of course (see Briner & Walshe, 2015; Nielsen, Randall, et al., 2010). However, their discussion here is beyond the scope of this paper. In addition, given the focus in this study on controlled interventions, the quantitative nature of a meta‐analysis and The Cochrane Collaboration's guidelines which have been followed throughout, we consider the ‘Risk of Bias’ tool to be an appropriate means for providing a snapshot of study quality and enabling the results to be compared with meta‐analyses following a similar protocol. It was also not possible to conduct a meta‐analytic moderator analysis based on quality criteria, because of the inconsistent and limited number of studies which reported such data. For example, eight studies did not report a drop‐out rate, and where rates had been reported, five reported a single, exact value, three reported a value per group (intervention and control) and two reported a value for the control group only. Conducting a moderator analysis on this data would therefore involve excluding nearly 50% of the studies and making several assumptions about the data available, rendering the results highly dubious at best.

## Results

### Demographics

Double coding of all 20 included studies revealed that 14 examined the effect of an intervention on overall work engagement between baseline (Time 1; T1) and post‐intervention (Time 2; T2) or between baseline and follow‐up (Time 3; T3). These formed the basis of the core meta‐analysis investigating the overall effect of interventions on work engagement (*N* = 3692). Ten measured vigour (*N* = 1501), nine measured dedication (*N* = 946) and six measured absorption (*N* = 732). Study sample sizes ranged between 45 (Carter, 2008) and 612 (Vuori et al., 2012). The gender distribution between samples varied considerably (~25% –96% male for those studies which provided this data, *k* = 15), as did participant age at baseline (~27–61 years).

Four of the fourteen studies included in the core meta‐analysis focused on interventions to increase personal resources, two focused on increasing job resources, four focused on health promotion and four focused on leadership training (Table 1). Two of these studies conducted an intervention entirely online, one conducted a face‐to‐face, individual intervention, eight conducted group based interventions and three employed both group‐based and individual strategies. Four of the 14 were conducted in public organisations, five in private organisations and five did not provide this data. Eight of the 14 studies were randomised and five employed the intention‐to‐treat principle. 5The intention‐to‐treat principle refers to an analysis in which every participant who was initially randomised into a study at baseline is included in the analysis whether or not they withdrew or adhered to the intervention (Fisher et al., 1990). Finally, nine were published journal articles, three were unpublished doctoral dissertations and one was a report which detailed two of the included studies. Please see Table 1 for an overview of the characteristics of each of the studies.

**Table 1:** Summary of the core characteristics of the twenty included studies in the meta‐analyses.

Reference	Doc^a^ type	Setting	Design^b^	Duration (T1–T2; T3)^c^	Intervention type	Intervention style	Core intervention components
Aikens et al., 2014	P	USA, chemical company	R	7 weeks; 6 months	Health promotion	Group	Virtual mindfulness sessions, homework, progress tracking survey, e‐coaching
Angelo & Chambel, 2013	P	Portugal, fire service	R	4 months	Leadership training	Online and group	Three day stress management workshop for supervisors with educational and action components
Biggs, 2011	T	Australia, police service	NR	7 months	Leadership training	Group	Participatory action research—six workshops involving psychoeducation and cognitive behavioural therapy based skills training (topics included stress, social support, career development and work–life balance)
Biggs et al., 2014	P	Australia, police service	NR	7 months;	Leadership training	Group	Action‐learning workshops over five days, involving education, a practical project and individual coaching
Calitz, 2010	T	South Africa, social work	NR	32 h; 1 month	Health promotion	Group	Two days of group sessions covering work engagement, job satisfaction, burnout, stress etc.
Carter, 2008	T	Australia, financial services	RM	5 months; 8 months	Personal resource building	Group	‘Forum theatre’ (vicarious learning), ‘rehearse for Reality’ (role play), ‘entertainment education’ (DVDs of ‘actors’ participating in role plays)
Chen et al., 2009	P	Israel, unknown organisation	CR (units)	2 weeks; 10 weeks	Personal resource building	Group	Conservation of resources (COR) intervention—five days of technical training before a new computer system was installed
Cifre et al., 2011	P	Spain, manufacturing	NR	6 months; 9 months	Job resource building	Individual	Action‐research approach to redesigning supervisor's role, increasing employee awareness of job training completed, job training
Coffeng et al., 2014	P	Finland, financial services	R	6 months; 12 months	Job resource building	Group	Combined social and environmental intervention—‘Vitality in Practice’ zones created (e.g. coffee zones, meeting zones), group motivational interviewing by team leaders, physical activity and relaxation encouraged
Hengel et al., 2012	P	The Netherlands, construction sites	CR	3 months; 12 months	Health promotion	Group and individual	Individual training sessions to lower physical workload, rest‐break tool, group empowerment sessions
Imamura et al., 2015	P	Japan, IT company	RCT	3 months; 6 months	Health promotion	Online and individual	Six week online cognitive behavioural therapy programme, voluntary homework and feedback from a Clinical Psychologist
Kmiec, 2010	T	USA, manufacturing	NR	90 days	Leadership training	Group	Education, skills practice and self‐coaching via classroom teaching and online learning
Naruse et al., 2014	P	Japan, Community nursing	NR	6 months	Job resource building	Individual	Skill‐mix intervention—nurses offered an assistant for community visits
Ouweneel et al., 2013	P	The Netherlands, Various organisations	NR	8 weeks; 16 weeks	Personal resource building	Online, individual	Weekly assignments (e.g. goal setting)
Rigotti et al., 2014	G	Germany, various	NR	14 months; 20 months	Leadership training	Group	Lectures including work and health, co‐operation and goal‐setting, leader training, including observations of leaders, feedback and coaching
Rigotti et al., 2014	G	Sweden, unknown organisations	NR	14 months; 20 months	Leadership training	Group	As above
Sodani et al., 2011	G	Iran, welfare organisation	RMP	Unknown	Personal resource building	Group	Nine creativity learning group sessions focused on problem solving and perspective taking
Strijk et al., 2013	P	The Netherlands, two academic hospitals	R	6 months; 12 months	Health promotion	Group and individual	Personal coach, yoga and aerobics, free fruit
Van Berkel et al., 2014	P	The Netherlands, two research institutes	R	6 months; 12 months	Health promotion	Group and individual	Group mindfulness training, goal‐setting, homework, individual e‐coaching, free fruit and veg snacks, buddy system, supporting materials (e.g. web page, logbook)
Vuori et al., 2012	P	Finland, various organisations	R	1 week; 7 months	Personal resource building	Group	Active learning, role playing, social modelling

a Type of document: P = Published in a peer reviewed journal; G = Grey literature; T = PhD thesis.

b Design: R = Randomised allocation at the individual level; CR = Cluster randomised allocation at the level of departments/units; RM = randomised matched groups; RMP = randomised matched pairs; NR = Non‐randomised allocation.

c Duration: length of time between the pre‐intervention measurement (T1) and the first post‐intervention measurement (T2). T3 indicates the length of time between the pre‐intervention measurement and a further, follow‐up measurement for those studies which collected this data.

### Overall effectiveness of work engagement interventions

The meta‐analysis revealed a small, positive, but reliable effect on work engagement (Hedges *g* = 0.29, 95%‐CI = 0.12–0.46), vigour (Hedges *g* = 0.95, 95%‐CI = 0.49–1.41), dedication (Hedges *g* = 0.75, 95%‐CI = 0.36–1.14) and absorption (Hedges *g* = 0.78, 95%‐CI = 0.33–1.22, Table 2). The sustainability of these effects is unclear, with work engagement and vigour demonstrating larger effects immediately post‐intervention than at follow‐up, and dedication and absorption demonstrating the opposite (Table 3). It was not possible to statistically investigate whether significant differences existed between subgroups because of the non‐independence of studies included in each. As expected, the heterogeneity between the studies was large for all the analyses (Tables 2 and 3). To investigate the effect of time further, meta‐regression, treating time as a continuous predictor, was conducted on studies measuring overall work engagement. Results revealed no moderation effect, suggesting that the effect size of interventions did not vary according to the duration between study measurements 6One study could not be included because of insufficient information indicating the duration between study measurements (Sodani et al., 2011). (*k* = 13, *n* = 3,652, *β* ≤ 0.01, SE ≤ 0.01, *p* = 0.85).

**Table 2:** Meta‐analytic results for the effects of interventions on work engagement, vigour, dedication and absorption.

Outcome	*k*	*n* (int)	*n* (con)	Intervention effects			*p*	Heterogeneity within each subgroup			
				*g*	SE	95%‐CI		Q	*df*	*p*	*I^2^*
Absorption^a^	7	360	372	0.78	0.23	0.33–1.22	0.00	43.45	6	0.00	86.19
Dedication^b^	10	458	488	0.75	0.20	0.36–1.14	0.00	62.52	9	0.00	85.60
Vigour^c^	11	708	793	0.95	0.23	0.49–1.41	0.00	145.64	10	0.00	93.13
Work engagement^d^	14	1758	1934	0.29	0.09	0.12–0.46	0.00	55.84	13	0.00	76.72

*Notes*. *K* = number of studies included in the analysis; *n*(con) = number of participants in the control group; *n*(int) = number of participants in the intervention group; *g* = average effect size according to Hedges' *g*; *SE* = standard error of the average effect size; 95%‐CI, *LL‐UL* = the minimum and maximum limits of the 95% confidence interval; *Q* = statistical test used for the estimation of heterogeneity; *I^2^* = proportion of effect size variance that can be attributed to moderator variables (%).

a Studies included: Aikens et al., 2014; Biggs, 2011; Calitz, 2010; Carter, 2008; Coffeng et al., 2014; Hengel et al., 2012; Sodani et al., 2011.

b Studies included: Aikens et al., 2014; Angelo & Chambel, 2013; Biggs, 2011; Calitz, 2010; Carter, 2008; Cifre et al., 2011; Coffeng et al., 2014; Hengel et al., 2012; Kmiec, 2010; Sodani et al., 2011.

c Studies included: Aikens et al., 2014; Angelo & Chambel, 2013; Biggs, 2011; Calitz, 2010; Carter, 2008; Chen et al., 2009; Cifre et al., 2011; Coffeng et al., 2014; Hengel et al., 2012; Sodani et al., 2011; Strijk et al., 2013.

d Studies included: Biggs et al., 2014; Carter, 2008; Coffeng et al., 2014; Hengel et al., 2012; Imamura et al., 2015; Kmiec, 2010; Naruse et al., 2014; Ouweneel et al., 2013; Rigotti et al., 2014 (two studies: German and Swiss samples); Sodani et al., 2011; Strijk et al., 2013; Van Berkel et al., 2014; Vuori et al., 2012.

**Table 3:** Meta‐analytic results for the effects of interventions on work engagement, vigour, dedication and absorption, at post‐intervention (T2) and follow‐up (T3).

Time point	*k*	n (int)	N (con)	Intervention effects			*P*	Heterogeneity within each subgroup			
				*G*	SE	95%‐CI,		Q	*df*	*p*	*I* ^2^
Absorption											
T2^a^	6	310	296	0.92	0.28	0.38–1.47	0.00	40.92	5	0.00	87.78
T3^b^	4	276	257	0.67	0.33	0.19–1.33	0.04	30.33	3	0.00	90.11
Dedication											
T2^c^	9	408	412	0.83	0.23	0.37–1.28	0.00	62.20	8	0.00	87.14
T3^d^	4	276	257	0.84	0.39	0.08–1.60	0.03	38.83	3	0.00	92.27
Vigour											
T2^e^	9	636	646	0.77	0.21	0.36–1.18	0.00	76.03	8	0.00	89.48
T3^f^	6	548	578	1.05	0.35	0.37–1.74	0.00	113.57	5	0.00	95.59
Work engagement											
T2^g^	12	1343	1517	0.31	0.10	0.12–0.45	0.00	55.84	11	0.00	80.30
T3^h^	8	1396	1312	0.16	0.05	0.07–0.24	0.00	2.74	7	0.91	0.00

*Notes*. *k* = number of studies included in the analysis; *n*(con) = number of participants in the control group; *n*(int) = number of participants in the intervention group; *g* = average effect size according to Hedges' *g*; *SE* = standard error of the average effect size; 95%‐CI, *LL‐UL* = the minimum and maximum limits of the 95% confidence interval; *Q* = statistical test used for the estimation of heterogeneity; *I^2^* = proportion of effect size variance that can be attributed to moderator variables; T2 = length of time between the pre‐ and post‐intervention measure; T3 = length of time between the pre‐intervention measure and a further, follow‐up measure (following the post‐intervention measure), for those studies which collected this data.

a Studies included: Aikens et al., 2014; Biggs, 2011; Calitz, 2010; Carter, 2008; Hengel et al., 2012; Sodani et al., 2011. Range of time between post‐intervention (T2) measurements (T2 range): 32 h–7 months after baseline (T1).

b Studies included: Aikens et al., 2014; Calitz, 2010; Coffeng et al., 2014; Hengel et al., 2012. Range of time between follow‐up (T3) measurements (T3 range): 1 month–12 months after baseline.

c Studies included: Aikens et al., 2014; Angelo & Chambel, 2013; Biggs, 2011; Calitz, 2010; Carter, 2010; Cifre et al., 2011; Hengel et al., 2012; Kmiec, 2010; Sodani et al., 2011. T2 range: 32 h–6 months

d Studies included: Aikens et al., 2014; Calitz, 2010; Coffeng et al., 2014; Hengel et al., 2012. T3 range: 1 month–12 months.

e Studies included: Aikens et al., 2014; Angelo & Chambel, 2013; Biggs, 2011; Calitz, 2010; Carter, 2008; Cifre et al., 2011; Hengel et al., 2012; Sodani et al., 2011; Strijk et al., 2013. T2 range: 32 h–6 months.

f Studies included: Aikens et al., 2014; Calitz, 2013; Chen et al., 2009; Coffeng et al., 2014; Hengel et al., 2012; Strijk et al., 2013. T3 range: 1 month–12 months.

g Studies included: Biggs et al., 2014; Carter, 2008; Coffeng et al., 2014; Hengel et al., 2012; Imamura et al., 2015; Naruse et al., 2014; Ouweneel et al., 2013; Rigotti et al., 2014 (two studies: German and Swiss samples); Sodani et al., 2011; Strijk et al., 2013; Van Berkel et al., 2014. T2 range: 8 weeks–14 months.

h Studies included: Coffeng et al., 2014; Hengel et al., 2012; Imamura et al., 2015; Rigotti et al., 2014 (two studies: German and Swiss samples); Strijk et al., 2013; Van Berkel et al., 2014; Vuori et al., 2012. T3 range: 1 week–18 months.

### Intervention type as a moderator of intervention effectiveness

To determine whether intervention type is associated with intervention effectiveness, the Q test, based on analysis of variance (Borenstein, Hedges', Higgins, & Rothstein, 2009), and assuming that intervention effect varies randomly between studies, was used to identify significant differences between groups. No significant differences in effect size were observed between intervention types on work engagement, *Q*(3) = 3.18, *p* = 0.36 (Table 4), indicating that intervention type is not a moderator. Study variance within subgroups was still large, therefore further moderator analyses were conducted (Table 4). These moderator variables were investigated based on their inclusion in meta‐analyses in related fields (e.g. intervention style and design, Maricuţoiu et al., 2016) and observed differences in study characteristics (e.g. organisation type, time between measurement points). A statistically significant difference between subgroups was observed for intervention style, *Q*(3) = 10.89, *p* = 0.01, with medium to large positive effects for group interventions (Hedges *g* = 0.51, 95%‐CI, 0.12–0.90, *p* = 0.01). Given that the ‘individual’ category contained only one study (Naruse et al., 2014), limiting the conclusions that can be drawn, a sensitivity analysis was conducted with this study removed. This reduced the significance to borderline, *Q*(3) = 4.641, *p* = 0.10. Further studies are needed within the ‘individual’ category to clarify these results. Non‐statistically significant differences between subgroups were observed for all other moderators (private versus public organisations, randomised versus non‐randomised, and adjusted for covariates such as age and gender versus not adjusted; results available on request), although all of the effects observed were positive and reliable. According to these results, there were no significant moderators of work engagement interventions, only a borderline significant moderator of intervention style. It should be noted, however, that the limited number of studies in subgroups may have resulted in power too low to detect an effect, decreasing the robustness of the results.

**Table 4:** Results of moderator analyses investigating the effect of differences in intervention study and methodological characteristics on work engagement.

Outcome	*k*	*n* (int)	*n* (con)	Intervention effects			*p*‐value	Heterogeneity within each subgroup			
				*g*	SE	95%‐CI		Q	*df*	*p*	*I^2^*
Intervention type											
Health promotion	4	806	815	0.14	0.06	0.02–0.26	0.03	4.24	3	0.24	29.23
Job resources	2	88	173	0.40	0.22	−0.04–0.84	0.08	2.84	1	0.09	64.75
Leadership training	4	371	337	0.14	0.08	−0.01–0.30	0.07	1.72	3	0.63	0.00
Personal resources	4	493	609	1.00	0.61	−0.20–2.20	0.10	42.08	3	0.00	92.87
					Heterogeneity between			3.18	3	0.36	
Intervention style											
Group	8	828	797	0.51	0.20	0.12–0.90	0.01	43.28	7	0.00	83.83
Group and individual	3	536	479	0.07	0.06	−0.05–0.20	0.26	0.98	2	0.61	0.00
Individual	1	38	97	0.63	0.19	0.25–1.01	0.00	0.00	0	1.000	0.00
Online and individual	2	356	561	0.17	0.11	−0.05–0.38	0.14	2.27	1	0.13	55.88
					Heterogeneity between			10.89	3	0.01	
Type of organisation											
Private	5	535	573	0.24	0.09	0.07–0.41	0.01	5.99	4	0.20	33.21
Public	4	549	676	0.12	0.10	−0.03–0.38	0.90	8.41	3	0.04	64.31
					Heterogeneity between			1.76	2	0.42	

*Notes*. *k* = number of studies included in the analysis; *n*(con) = number of participants in the control group; *n*(int) = number of participants in the intervention group; *g* = average effect size according to Hedges' *g*; *SE* = standard error of the average effect size; 95%‐CI, *LL‐UL* = the minimum and maximum limits of the 95% confidence interval; *Q* = statistical test used for the estimation of heterogeneity; *I^2^* = proportion of effect size variance that can be attributed to moderator variables (%).

Publication bias was not detected, (Rosenthal's (1991) Fail‐Safe *N* = 127), and Duval and Tweedie's (2000) Trim and Fill method suggested that three studies would need to fall on the right hand side of the mean effect size to make a funnel plot symmetric. Assuming a random effects model, the new imputed mean effect size would be, Hedges *g* = 0.40, 95%‐CI = 0.22–0.57 (previously, Hedges' *g* = 0.29, 95%‐CI = 0.12–0.46). These results suggest that the effect size estimates from the observed studies were unbiassed and robust. However, the quality assessment using The Cochrane Collaboration's (2011) ‘Risk of Bias’ tool revealed all studies to be at high risk of bias. This should be taken into account when interpreting the results.

## Discussion

### The effectiveness of work engagement interventions

This study aimed to address two research questions: (i) whether work engagement interventions are effective; and (ii) whether intervention type is associated with intervention effectiveness. The systematic literature search revealed 20 papers which met the inclusion criteria and could be included in the meta‐analyses. The meta‐analytic results demonstrated a positive, small, significant, effect on work engagement and each of its three sub‐components, vigour, dedication and absorption. This suggests that interventions aimed at increasing resources in the work environment and improving well‐being can improve employees' work engagement, in accordance with the JD‐R model. This effect was observed across a range of countries, organisational settings, industries and participant characteristics, suggesting generalisability and thus the benefit of work engagement interventions to organisations globally.

The slightly larger positive effects observed for the sub‐components compared to overall work engagement may reflect previous results which suggest that the psychometric properties of a three‐factor model are superior to those of a unidimensional, one factor model (Schaufeli et al., 2006). This raises further questions over the meaning of engagement and how best to operationalise it, and could suggest that it is more effective to direct interventions at increasing one or other of the sub‐components rather than overall work engagement. Analysing the effectiveness of different aspects of these interventions (e.g. skills sessions, coaching, homework) could take work engagement research further still, and contribute to an evidence‐based discussion of how and why interventions work.

Meta‐analysis revealed ambiguous results regarding the sustainability of effects, with stronger effects for vigour immediately post‐intervention than at follow‐up, and the opposite being true for dedication and absorption. Meta‐regression, however, did not reveal a significant effect of time on studies measuring overall work engagement. These results could suggest lasting effects of interventions and are not dissimilar from results found by other studies in related areas which have investigated the effect of intervention duration on outcomes. For example, Maricuţoiu et al. (2016) investigated the effectiveness of controlled interventions on employees' burnout and found that average effect sizes were similar across different measurement time points. More studies are needed, however, which measure work engagement and its sub‐components at various points following interventions, to clarify these results.

A moderator analysis did not reveal a significant effect of intervention type on the effectiveness of work engagement interventions, meaning that we cannot reject the null hypothesis of no difference between intervention types. This suggests that success is not affected by the focus of the intervention. This could be because of indirect effects on job and personal resources, as well as well‐being. For example, an intervention designed to directly increase personal resources could accordingly increase an individual's sense of self‐esteem, competence and experience of positive emotions, broadening the number and type of thoughts and actions that come to mind, in accordance with broaden‐and‐build theory (Fredrickson, 2001). This could in turn lead to individuals searching out opportunities, crafting their own jobs and increasing their job resources and sense of well‐being.

Alternatively, the lack of effects observed for intervention type may have been because of the high heterogeneity between the studies within each subgroup. This has been observed in meta‐analyses in related areas (e.g. Richardson & Rothstein, 2008) and prevents a meaningful comparison between the interventions or generalisation of the results. It also suggests that other factors may explain the results and account for why a significant effect of intervention type was not found. In particular, included studies varied in terms of design and content; thus, it is impossible to determine whether the results observed were because of these, or indeed other, variables not measured by the interventions. Furthermore, poor intervention implementation may have decreased the ability of meta‐analyses to detect an effect. A general discussion about this follows shortly.

To explore the high level of heterogeneity between studies further, another moderator analysis involving intervention style was conducted, which revealed a significant effect with a medium to strong positive effect for group interventions. This result was reduced to borderline significance when the ‘individual’ category, containing a single study, was removed. Further studies are needed which have been conducted on a one‐to‐one, individual basis in order to increase the robustness of the results and draw conclusions about the effect of intervention style for improving work engagement. Nevertheless, these results suggest that intervention style may be a more important moderator than intervention type, and with further studies, more differences may be discovered which build on them.

A possible explanation for the strength of the effect for group interventions is that they effectively influence certain work engagement antecedents, such as social support and influence in decision‐making. In accordance with the JD‐R model, an increase in these resources could boost work engagement and protect against negative outcomes such as burnout and stress. Research which has investigated the effectiveness of group interventions to manage stress offers support for this explanation. For example, Nielsen, Randall, and Albertsen (2007) found that employees who were able to work together to influence and decide the content of stress management interventions reported increased job satisfaction and improved working conditions and behavioural stress symptoms. Furthermore, Park et al. (2004) found that a group, problem‐solving intervention was positively related to organisational social climate and interactions with colleagues and supervisors, and a systematic review found that 11 group, organisational‐level occupational health interventions (eight of which were controlled) were associated with positive outcomes, out of a total of 18 studies (Egan et al., 2007).

The studies discussed above suggest that participating in a group intervention, with the opportunity to talk to colleagues, develop personal relations and work skills, and voice an opinion, enables individuals to increase job resources such as social support and influence in decision‐making, leading to positive outcomes. Because the JD‐R model predicts that increases in such resources activate the motivational pathway, it follows that group interventions which focus on increasing these resources should lead to increased work engagement and well‐being in participants. Nielsen (2013) applies Social Identity Theory (SIT) to explain how group interventions may enhance participants' resources. Nielsen proposes that individuals participating in a group intervention build a sense of identity with their group. Being a member of this in‐group provides individuals with the opportunity to work with others towards a common goal, such as improving an aspect of the work environment or solving a particular work‐related problem. This could increase the job resource, social support (Nielsen, 2013), as well as enabling individuals' needs for a sense of belonging, purpose and meaning to be met, positively affecting well‐being (Haslam, Jetten, Postmes, & Haslam, 2009) and work engagement. Group interventions may therefore be a productive focus for future work engagement interventions, and we encourage further research to further understand the effectiveness of group interventions.

Further meta‐analytic moderator and sensitivity analyses revealed no statistically significant differences between groups for organisation type (private vs public), study design (randomised vs non‐randomised) or degree of statistical control (results adjusted for covariates vs not adjusted). These results suggest that the effectiveness of work engagement interventions may be generalized across private and public organisations, and are not affected by whether studies are randomised or not. This latter conclusion supports a growing body of evidence which suggests moving away from applying the traditional ‘gold standard’ randomised controlled design in all settings and circumstances towards an assessment of the most appropriate design for the individual context of the study (e.g. Briner & Walshe, 2015; Nielsen, Taris and Cox, 2010). Proponents of this view suggest applying appropriate referent groups, whether randomised or not.

### Limitations

There were a number of limitations to this study. The first pertains to the low sample size of studies included in the moderator analyses, reducing the power of the analyses and limiting the extent to which conclusions can be drawn. However, small numbers are not unusual in organisational psychology research (e.g. Maricuţoiu et al., 2016; Richardson & Rothstein, 2008), and sensitivity analyses did not indicate biassed results. The second is the potential for bias because of misclassification of studies. Most studies contained a number of different intervention components and involved a number of different styles of delivery (Table 1). Erroneous judgements could therefore have been made; however, each study was double coded by an independent researcher according to a detailed coding guide (see Supplemental file 2), and agreement reached 100% for every piece of data extracted, limiting this possibility.

Third, the intervention studies which our search revealed almost exclusively relied on studies measuring engagement using the UWES. This does not reflect the fractured nature of the field regarding the meaning and measurement of engagement, but it does reflect the dominance of the measure. Its dominance, however, does not make it the ‘best’ measure, and its validity and reliability have most recently been questioned by Byrne et al. (2016), who found that the UWES overlaps with other job attitudes such as stress, job performance, organisational commitment and burnout. Its dominance in our study could potentially be viewed as an advantage, as results were able to be more meaningfully synthesised and interpreted than if a plethora of different measures and definitions of engagement had been analysed together. Fourth, it is possible that regression to the mean explains the significant moderator effect of intervention style. Therefore, scores on work engagement for those in group interventions may have been further from the average population score pre‐intervention, and nearer to the population mean post‐intervention. The difference between these scores in comparison to those for the other subgroups may have led to the significant subgroup effect observed.

Fifth, meta‐analyses are not able to control for differences in how well interventions are implemented and attended by participants who are motivated to take part. Nielsen et al. (2007) analysed data from 11 stress management interventions and found that participants' perceptions of interventions (i.e. whether participants considered the intervention of high quality and able to bring about sustained change) were related to intervention outcomes such as job satisfaction, changes in working conditions and behavioural stress symptoms. This indicates the importance of intervention implementation and participant buy‐in to the overall success of interventions. Poor intervention implementation may therefore account for the small overall effect observed for work engagement interventions. Some work engagement intervention studies provided information which supports this. For example, Strijk et al. (2013) found that significant positive effects were observed for those who achieved above average attendance at sessions, and Coffeng et al. (2014) noted that if compliance had been higher, their intervention may have been more effective. Problems cited by individual studies included difficulty scheduling workshops because of the geographical distance between participant teams (Rigotti et al., 2014), lack of time (e.g. Rigotti et al., 2014; Strijk et al., 2013), lack of support from managers (e.g. Rigotti et al., 2014; Strijk et al., 2013), inconvenient location or timing of interventions (e.g. Strijk et al., 2013), relocation of participants (Rigotti et al., 2014) and sickness absence (e.g. Hengel et al., 2012; Strijk et al., 2013).

In relation to study implementation, several studies also reported adverse factors which were beyond the control of the researchers and could not have been predicted, but which may have impacted the results. These included organisational restructuring (e.g. Rigotti et al., 2014; Van Berkel et al., 2014), a proposed corporate merger (Carter, 2008), economic downturn and job insecurity (e.g. Carter, 2008; Hengel et al., 2012), redundancy (Aikens et al., 2014), a pay restructure (Kmiec, 2010) and a plant fire which affected the number of hours of physical labour that employees were working (Kmiec, 2010). Although few studies overall thoroughly assessed implementation (*k* = 3), it seems plausible that an intervention which was delivered as planned, was well attended, and actively engaged and motivated participants would be more successful. Indeed, several researchers promote the inclusion of an implementation evaluation as a matter of course in order to go beyond simply stating whether or not an intervention was effective, and discover the reasons for *why* and *how* an intervention was effective (e.g. Briner & Walshe, 2015; Nielsen, Taris and Cox, 2010). Including such evaluations is one way of taking work engagement intervention research forward.

Finally, in relation to implementation, the variable and sometimes poor response and attrition rates (18–94% and 5–88%, respectively) affected the degree to which results could be generalized. Indeed, some studies found demographic or outcome differences between those who dropped out and those who did not (e.g. Ouweneel et al., 2013; Vuori et al., 2012), suggesting bias. Additionally, some of the sample sizes were small (e.g. Angelo & Chambel, 2013; Rickard et al., 2012), possibly resulting in statistical power too low to detect an effect. Many of the issues with implementation cited here could be somewhat mitigated by a pre‐assessment of the organisation's suitability for an intervention and the readiness to change of participants (Briner & Walshe, 2015; Nielsen, Randall, et al., 2010). Furthermore, carefully planning and nurturing researcher–organisation relations is likely to be essential in order to pave the way for more successfully implemented interventions.

### Implications for future research and practice

This is the first systematic review and meta‐analysis to evaluate the effectiveness of controlled interventions to increase work engagement and thus contributes uniquely to the developing evidence‐base. In particular, it details the development of a novel taxonomy of work engagement interventions which researchers can use to develop streams of research in this area. Importantly, this taxonomy can be built upon as the field progresses and could help identify further factors which moderate the effectiveness of interventions, and are thus important for building work engagement. In this way, this review engages conversation around how and why interventions work, and stimulates the advancement of work engagement theory.

This review also highlights the need for more work engagement interventions, and tentatively suggests that group interventions could be an effective way of taking work engagement intervention research forward. Furthermore, it supports the argument that the success of researcher–organisation relationships is crucial to intervention implementation, with lack of support from top management being cited by several participants as to why they were unable or unmotivated to take part. Researchers would therefore be advised to carefully negotiate and manage relationships with their partner organisation(s) throughout the intervention process. Finally, our research supports the view that including a detailed evaluation of the success of intervention implementation would go some way towards furthering understanding of how and why interventions work and we would urge researchers to conduct one as a matter of course.

## Conclusion

Three conclusions can be drawn from these results. First, the meta‐analysis demonstrated that interventions to increase work engagement in organisations may be effective. Second, the tentatively significant meta‐analytic moderator analysis for intervention style demonstrated a medium to large effect of group interventions, suggesting the benefit of working in groups for increasing resources, work engagement and well‐being. Developing group interventions could therefore be an effective way of taking work engagement intervention research forward. Third, all the analyses indicated large heterogeneity, suggesting other important moderators and subgroups. However, at present, the small number of studies precludes an investigation of these; hence, it is impossible at this stage to meaningfully compare interventions and determine which characteristics may be more or less important for their success. It is hoped that the results from this first meta‐analysis of work engagement interventions will stimulate discussion amongst academics and practitioners and help to inform the direction of future research and practice aimed at building and sustaining work engagement.

## Supporting information

Supplemental file 1. Database search strategies for systematically identifying work engagement interventions

Supplemental file 2. Coding guide for coding work engagement intervention studies
