# Supplementary material for: Building work engagement: A systematic review and meta‐analysis investigating the effectiveness of work engagement interventions
Source: J Organ Behav. 2016 Dec 13;38(6):792–812. doi: 10.1002/job.2167 (PMC5516176; doi:10.1002/job.2167)
Supplement: Supplementary file 1 — Supplemental file 1. Database search strategies for systematically identifying work engagement interventions [file JOB-38-792-s001.docx]

**Supplemental file 1: Database search strategies for systematically**

**identifying work engagement interventions**

*NOTE:* Search dates, number of hits and number of relevant hits, based on abstracts and titles only, are included with the search terms for each database below. These searches were also conducted on subsequent dates in order to check for additional sources which may have been published after previous searches were conducted. All searches were completed between May 2014 and May 2015. The final search terms used are included here.

**Search strategy for MEDLINE 11.08.14**

#1. Work engagement.ti

#2. Employee engagement.ti

#3. Job engagement.ti

#4. #1 OR #2 OR #3

#5. Group intervention.ti

#6. Individual intervention.ti

#7. Online intervention.ti

#8. Web intervention.ti

#9. Internet intervention.ti

#10. #5 OR #6 OR #7 OR #8

#11. #3 AND #9

*Note:* Experimenting with different words for ‘intervention’ did not reveal any more results which were not captured by using this search.

*Hits:* 17. 12 relevant (based on titles / abstracts).

**Search strategy for Web of Science Core Collection 21.08.14**

#1. Work engagement.topic

#2. Employee engagement. topic

#3. Job engagement. topic

#4. #1 OR #2 OR #3

#5. Group intervention. topic

#6. Individual intervention. topic

#7. Online intervention. topic

#8. Web intervention.topic

#9 Internet intervention.topic

#10. #5 OR #6 OR #7 OR #8 OR #9

#11. #4 AND #10

*Hits:* 409. 30 relevant (based on titles / abstracts).

**Search strategy for Scopus 19.08.14**

Search 1

(TITLE-ABS-KEY(work OR employee OR job) AND TITLE-ABS-KEY(group OR individual OR online OR web OR internet) AND TITLE-ABS-KEY(intervention OR compar*) AND TITLE-ABS-KEY(engagement)) AND (EXCLUDE(EXACTKEYWORD, "Adolescent") OR EXCLUDE(EXACTKEYWORD, "Students") OR EXCLUDE(EXACTKEYWORD, "Child") OR EXCLUDE(EXACTKEYWORD, "Adolescent") OR EXCLUDE(EXACTKEYWORD, "Students") OR EXCLUDE(EXACTKEYWORD, "Child")) AND (EXCLUDE(EXACTKEYWORD, "Education") OR EXCLUDE(EXACTKEYWORD, "Education")) AND (EXCLUDE(EXACTKEYWORD, "Aged") OR EXCLUDE(EXACTKEYWORD, "Mental disease") OR EXCLUDE(EXACTKEYWORD, "Mental health") OR EXCLUDE(EXACTKEYWORD, "Young Adult") OR EXCLUDE(EXACTKEYWORD, "Aged") OR EXCLUDE(EXACTKEYWORD, "Mental disease") OR EXCLUDE(EXACTKEYWORD, "Mental health") OR EXCLUDE(EXACTKEYWORD, "Young Adult"))

*Hits* 831.

Excluded ‘Review’: hits 739

Limited to ‘work engagement’ and ‘engagement’ keywords: hits: 64. 6 relevant (based on titles / abstracts).

This search was repeated on 18.09.14, revealing identical results.

Search 2 - **18.09.14**

((TITLE-ABS-KEY("work engagement") OR TITLE-ABS-KEY("employee engagement") OR TITLE-ABS-KEY("job engagement"))) AND ((TITLE-ABS-KEY(group W/5 intervention) OR TITLE-ABS-KEY(individual W/5 intervention) OR TITLE-ABS-KEY(web W/5 intervention) OR TITLE-ABS-KEY(internet W/5 intervention) OR TITLE-ABS-KEY(online W/5 intervention)))

Hits: 19. Relevant: 16 (some were the same as those found in search 1)

**ProQuest Dissertations and Theses 15.08.14**

Databases covered:

COS Conference Papers Index

ProQuest Dissertations & Theses: UK & Ireland

ProQuest Dissertations & Theses A&I

Search 1:

(work-engagement-intervention OR employee-engagement-intervention OR job-engagement-intervention) AND la.exact (‘English’).

Hits: 9. 1 relevant (based on titles / abstracts).

Search 2: **15.08.14 and 18.09.14**

(work-engagement OR employee-engagement OR job-engagement) AND (group-intervention OR individual-intervention OR online-intervention OR web-intervention OR internet-intervention) AND la.exact (‘English’).

Hits 128. 6 relevant (based on titles / abstracts).

**International Bibliography of the Social Sciences (IBSS) 19.09.14**

(work engagement OR employee engagement OR job engagement (Anywhere)) AND intervention (anywhere)

Hits: 8. None relevant (based on titles / abstracts).

**Trove – Australian theses 19.09.14**

Search 1

(‘work engagement’ OR ‘employee engagement’ or ‘job engagement’) AND ‘intervention’

*Hits:* 34. 4 relevant (based on titles / abstracts).

**Thesis Canada Portal (Canadian etheses)**

Search 1 **19.09.14**

(‘work engagement’ OR ‘employee engagement’ or ‘job engagement’) AND ‘intervention’)

0 hits.

**Google Scholar 15.08.14**

Search 1

Advanced search: All words in title of article: employee engagement intervention

*Hits:* 7. 3 relevant (based on titles / abstracts).

Search 2

Advanced search: All words in title of article: work engagement intervention

Hits: 27. 10 relevant (based on titles / abstracts).

*NOTE:* job engagement intervention (all in title) returned no hits
