# Supplementary material for: Building work engagement: A systematic review and meta‐analysis investigating the effectiveness of work engagement interventions
Source: J Organ Behav. 2016 Dec 13;38(6):792–812. doi: 10.1002/job.2167 (PMC5516176; doi:10.1002/job.2167)
Supplement: Supplementary file 2 — Supplemental file 2. Coding guide for coding work engagement intervention studies [file JOB-38-792-s002.docx]

**Supplemental file 2: Coding guide for coding work engagement intervention studies**

| **No.** | **Variable** | **Codes** | **Definition & examples** |
| --- | --- | --- | --- |
| 1 | Study number | 1, 2, 3…etc | - Each study should have an individual number assigned to it which is the same as that assigned by the first coder - If a primary article / document contains two or more sets of results from **independent** studies or samples (e.g. from two different countries), these should be treated separately and given their own identifying number. This means that each set of results can be included separately in meta-analyses |
| 2 | Reference | First author & year | - Name each paper by the first author, followed by the year in which the paper / document was published / made available - If two independent studies emerge from a single paper, add a code following the author name to distinguish between them (e.g GER for a study occurring in Germany, and SWE for a study occurring in Sweden) |
| 3 | Type of literature | Published | - Primary articles published in peer-reviewed journals |
|  |  | Thesis | - Either PhD or MA theses |
|  |  | Grey | - Reports / documents which are not published in peer-reviewed journals and which are not theses e.g. government reports, health organisation reports, conference papers etc. |
| 4 | Design | Randomised | - Study in which participants are randomly allocated to groups, including studies in which pairs of participants are matched and then randomised - If an author states randomisation has occurred, but it is unclear how this has occurred, discuss this issue in the study’s respective ‘risk of bias’ table |
|  |  | Cluster randomised | - Including randomisation at department level, ‘unit’ level etc but NOT randomised matched pairs |
|  |  | Non-randomised | - No evidence of randomisation at any level |
| 5 | Industry | Nursing | - Categorise the organisations according to which type of industry they best represent. |
|  |  | Financial services |  |
|  |  | Construction |  |
|  |  | Research institutes |  |
|  |  | Chemical company |  |
|  |  | Manufacturing company |  |
|  |  | Fire service |  |
|  |  | Social work service |  |
|  |  | Police service |  |
|  |  | Welfare organisation |  |
|  |  | Various |  |
|  |  | Not specified |  |
| 6 | Private or public organisation? | Public | - Organisations which are subsidised by the government e.g. hospitals, university research institutions - Use this definition for organisations in other countries which would be classed as ‘public’ in the UK |
|  |  | Private | - Organisations which are not subsidised by the government e.g. banks - Use this definition for organisations in other countries which would be classed as ‘private’ in the UK |
|  |  | Unknown | - Use this for studies which involve organisations across the public or private sector, preventing a code being assigned, or when there is not enough information to categorise an organisation |
| 7 | Country | Exact name of country | - Code the country exactly as found in the article (i.e. with the name of that country). - If a state or continent is given but no country, where possible deduct the country from other information given in the article / document, otherwise code as ‘unknown’. |
|  |  | Various | - If several countries are involved, state ‘various’, unless the samples involved in each country are counted as separate studies (see ‘study number’ above) |
|  |  | Unknown | - If a state or continent is given but no country, where possible deduct the country from other information given in the article / document, otherwise code as ‘unknown’. |
| 8 | Type of intervention 1 | Job resource building | **Definition:** In accordance with the Job-Demands Resources model (JD-R; Bakker and Demerouti, 2007; 2008), job resources refer to physical, social or organisational aspects of the job (e.g. feedback, social support, development opportunities) that can reduce job demands (e.g. workload, emotional and cognitive demands), help employees to achieve work goals and stimulate personal learning and development. Thus, interventions which build job resources may focus on changing aspects of:   - the physical environment e.g. redesigning physical layout of offices; - the social environment e.g. increasing supervisor & colleague support; - the resources of the individual e.g. job crafting interventions - systemic systems e.g. implementing an internal IT system   **NB:** Coding decisions may depend on the outcomes which have been collected; if the outcomes measured primarily reflect job resources, code the intervention as such. In addition, **code each intervention group within a study separately** |
|  |  | Personal resource building | **Definition:** Personal resources refer to ‘positive self-evaluations that are linked to resiliency and refer to individuals’ sense of their ability to control and impact upon their environment successfully’ (Bakker & Demerouti, 2008 p.5). These include, but are not limited to, self-esteem, self-efficacy, resilience and optimism. Interventions which aim to increase personal resources may therefore include:   - programmes to build self-efficacy, career self-efficacy, resilience or psychological capital - empowerment programmes - programmes to build on ‘strengths’ e.g. gratitude, kindness, curiosity   **NB:** Coding decisions may depend on the outcomes which have been collected; if the outcomes measured primarily reflect personal resources, code the intervention as such. In addition, **code each intervention group within a study separately** |
|  |  | Health promotion | **Definition:** Typically, ‘Worksite Health Promotion’ (WHP) interventions aim to promote positive outcomes such as work engagement, work-related wellbeing, and employee performance and productivity, whilst reducing negative outcomes such as absenteeism and presenteeism. For the purposes of this meta-analysis ‘health promotion’ interventions refer to all interventions which aim to improve positive outcomes or reduce negative outcomes, and thus include:   - stress reduction interventions - mindfulness based programmes - exercise programmes   **NB: Code each intervention group within a study separately** |
|  |  | Leadership training | **Definition:** Interventions conducted directly with leaders, managers and / or supervisors with the primary intention of impacting on these individuals’ leadership abilities and skills, and the secondary intention of impacting positively on their employees. Such interventions may include:   - educative workshops - self- and group- reflection   **NB:** Studies measuring outcomes in the employees of managers only, as opposed to the managers themselves, should still be coded as a leadership intervention. In addition, **code each intervention group within a study separately** |
| 9 | Style of intervention | Individual | - Conducted on a one-to-one, face-to-face basis |
|  |  | Online | - Conducted purely online, including both web based information resources and one-to-one e-coaching |
|  |  | Group | - Training conducted purely in groups, whether occurring ‘on-site’ or off. Includes training via webinars requiring participants in a particular study group to participate from the same location at the same time. |
|  |  | Individual & online | - Predominantly a mixture of ‘individual’ and ‘online’ training, as defined above. - There may be additional minor supporting elements e.g. buddy system |
|  |  | Individual and group | - Predominantly a mixture of ‘individual’ and ‘group’ as defined above - There may be additional minor supporting elements e.g. social support via social media such as a facebook page |
| 10 | No. intervention & control groups | 1, 1  1, 2  3, 1 | - State how many intervention and control groups there are e.g.’1, 1’ = 1 intervention group and 1 control group; 1, 2 = 1 intervention group and 2 control groups |
| 11 | Measure used | UWES-17 | - Full 17 item Utrecht Work Engagement Questionnaire used, in any language. |
|  |  | UWES-9 | - Abbreviated 9 item Utrecht Work Engagement Questionnaire used, in any language. |
|  |  | UWES-? | - Where the author has used an incomplete number of items for either the full or abbreviated version, state the number of items used overall e.g. UWES-6 items |
|  |  | Name of scale e.g. Shirom Vigour Scale | - Code all other validated, robust measures of work engagement by the name of that scale. Scales should contain an affective, cognitive and behavioural component for the study to be considered for inclusion |
|  |  | Unsure | - For studies which do not make it clear which scale of work engagement has been used |
| 12 | Outcomes | Work engagement | - Code each outcome which has been measured, pertaining to work engagement, on a separate row, so, for example, a study which measures all four possible outcomes will have 4 rows |
|  |  | Vigour |  |
|  |  | Dedication |  |
|  |  | Absorption |  |
| 13 | Waves | T1-T2 + length of wave | - Data collected at T1 (pre-intervention / baseline) and T2 (post-intervention). State the length of time between T1 and T2 exactly as stated in the study (e.g. 6 months, 90 days) - Code each outcome which has been measured at each time point on separate rows. For example, a study which has measured ‘vigour’ at T2 and T3 will have two rows for vigour, one stating T1-T2 in the ‘waves’ column, and one stating ‘T1-T3’.   **NB:** Do **NOT** count data collection which has occurred half way through an intervention as occurring at T2, as this data will not be included in a meta-analysis of the effect of interventions across T1-T2. Rather, to maintain consistency, count the post-intervention data collection time point as T2. |
|  |  | T1-T3 + length of time since T2 / post-intervention | - Data collected at T1 (pre- intervention) and at follow-up, but not necessarily at T2 (post-intervention). State the length of time between the end of the intervention and T3 exactly as stated in the study (e.g 2 months, 6 months) |
| 14 | Intention-to-treat principle followed? | ITT followed | - According to the Cochrane handbook, a ‘full ITT analysis’ refers to an analysis which includes ‘all participants who did not receive the assigned intervention according to the protocol as well as those who were lost to follow-up’(see Appendix 1 for more info). - In terms of the studies involved in this meta-analysis, **ITT analysis refers to an analysis using imputation for missing values**. It is based on the total number of randomized participants at baseline (see section 16.2.1 & 16.2.3 of the Cochrane Handbook for more info). - In accordance with The Cochrane Handbook, do not rely on authors’ judgements of whether an ITT analysis has been performed, but deduce the nature of the analysis from the information given |
|  |  | ITT not followed | - Any analysis which does not follow the principles of ITT analysis described above e.g. available case analysis, per-protocol analysis. |
|  |  | Both | - Both an ITT analysis, and an analysis which has not followed the principles of ITT, have been conducted |
| 15 | Analysis adjusted? | Yes – baseline values only | - An analysis which has been adjusted for covariates (e.g. age, gender, education etc.) and / or baseline differences on the outcome measure / dependent variables. Please note which adjustment has occurred. If both, please state ‘adjusted – both’ |
|  |  | Yes - covariates | - See above |
|  |  | No - Not adjusted | - Results which have not been adjusted for covariates e.g. raw means and SDs, F values which have not been adjusted etc   **NB:** If studies include both adjusted and non-adjusted results (e.g. raw means and SDS and adjusted F-values), it is necessary only to code the adjusted results |
